# The Use of a Mobile Laboratory Unit in Support of Patient Management and Epidemiological Surveillance during the 2005 Marburg Outbreak in Angola

**DOI:** 10.1371/journal.pntd.0001183

**Published:** 2011-05-24

**Authors:** Allen Grolla, Steven M. Jones, Lisa Fernando, James E. Strong, Ute Ströher, Peggy Möller, Janusz T. Paweska, Felicity Burt, Pedro Pablo Palma, Armand Sprecher, Pierre Formenty, Cathy Roth, Heinz Feldmann

**Affiliations:** 1 Special Pathogens Program, National Microbiology Laboratory, Public Health Agency of Canada, Winnipeg, Manitoba, Canada; 2 Department of Medical Microbiology, University of Manitoba, Winnipeg, Manitoba, Canada; 3 Department of Immunology, University of Manitoba, Winnipeg, Manitoba, Canada; 4 Department of Pediatrics and Child Health, University of Manitoba, Winnipeg, Manitoba, Canada; 5 Institut für Virologie, Philipps-Universität, Marburg, Hessen, Germany; 6 Special Pathogens Unit, National Institute for Communicable Diseases of the National Health Laboratory Service, Sandringham, South Africa; 7 Médecins Sans Frontières, Barcelona, Spain; 8 Médecins Sans Frontières, Brussels, Belgium; 9 World Health Organization, Geneva, Switzerland; University of Texas Medical Branch at Galveston, United States of America

## Abstract

**Background:**

Marburg virus (MARV), a zoonotic pathogen causing severe hemorrhagic fever in man, has emerged in Angola resulting in the largest outbreak of Marburg hemorrhagic fever (MHF) with the highest case fatality rate to date.

**Methodology/Principal Findings:**

A mobile laboratory unit (MLU) was deployed as part of the World Health Organization outbreak response. Utilizing quantitative real-time PCR assays, this laboratory provided specific MARV diagnostics in Uige, the epicentre of the outbreak. The MLU operated over a period of 88 days and tested 620 specimens from 388 individuals. Specimens included mainly oral swabs and EDTA blood. Following establishing on site, the MLU operation allowed a diagnostic response in <4 hours from sample receiving. Most cases were found among females in the child-bearing age and in children less than five years of age. The outbreak had a high number of paediatric cases and breastfeeding may have been a factor in MARV transmission as indicated by the epidemiology and MARV positive breast milk specimens. Oral swabs were a useful alternative specimen source to whole blood/serum allowing testing of patients in circumstances of resistance to invasive procedures but limited diagnostic testing to molecular approaches. There was a high concordance in test results between the MLU and the reference laboratory in Luanda operated by the US Centers for Disease Control and Prevention.

**Conclusions/Significance:**

The MLU was an important outbreak response asset providing support in patient management and epidemiological surveillance. Field laboratory capacity should be expanded and made an essential part of any future outbreak investigation.

## Author Summary

A mobile laboratory unit (MLU) was deployed to Uige, Angola as part of the World Health Organization response to an outbreak of viral hemorrhagic fever caused by Marburg virus (MARV). Utilizing mainly quantitative real-time PCR assays, this laboratory provided specific MARV diagnostics in the field. The MLU operated for 88 consecutive days allowing MARV-specific diagnostic response in <4 hours from sample receiving. Most cases were found among females in the child-bearing age and in children less than five years of age including a high number of paediatric cases implicating breastfeeding as potential transmission route. Oral swabs were identified as a useful alternative specimen source to the standard whole blood/serum specimens for patients refusing blood draw. There was a high concordance in test results between the MLU and the reference laboratory in Luanda operated by the US Centers for Disease Control and Prevention. The MLU was an important outbreak response asset providing valuable support in patient management and epidemiological surveillance. Field laboratory capacity should be expanded and made an essential part of any future outbreak investigation.

## Introduction

Marburg virus (MARV) is classified as members of the family *Filoviridae*, genus *Marburgvirus*, type species *Lake Victoria marburgvirus*. A single species has been described which includes several virus strains [1]. Today, the geographic distribution of MARV seems to primarily involve areas in East Africa within 500 miles of Lake Victoria, Zimbabwe, but also western Africa [2], [3]. MARV is of zoonotic nature with an as yet unidentified reservoir in nature, but with strong cumulative evidence that bats are involved in the zoonotic cycle [4], [5] as this has also been implicated for Ebola virus [6].

MARV is the causative agent of Marburg hemorrhagic fever (MHF), a disease that was first described in 1967 among laboratory workers in Germany and former Yugoslavia [7]–[9]. Until 1998, only sporadic MHF cases have occurred in Zimbabwe/South Africa (1975) and in Kenya (1980 & 1987) [10]–[12]. The first community-based MHF outbreak was reported in 1998–2000 from the Watsa/Durba region in the Democratic Republic of the Congo (DRC) [13], [14]. In 2004/2005 MARV first appeared in western Africa, Angola, causing to date the largest MHF outbreak on record [15], [16]. The latest MHF episodes involved 4 reported cases from western Uganda associated with a single mine (2007) [5], and two imported cases into the US and the Netherlands, who independently visited the same cave in Uganda (2008) [17], [18] (Table 1). In addition, three laboratory exposures, one of them fatal, have been reported [9], [19], [20].

**Table 1:** Documented outbreaks/episodes/cases of Marburg Hemorrhagic Fever (MHF).

Location	Year	Strain	Cases(Deaths)	Epidemiology
Germany/Yugoslavia [7]–[9]	1967	Ratayczak /Popp	32 (7)	Imported monkeys from Uganda source of primary human infections
Zimbabwe [10]	1975	Ozolin	3 (1)	Unknown origin; index case was infected in Zimbabwe (lethal), secondary cases in South Africa
Kenya [11]	1980	Musoke	2 (1)	Unknown origin; lethal index case was infected in western Kenya
Kenya [12]	1987	Ravn	1 (1)	Unknown origin; expatriate traveling in western Kenya
Democratic Republic of the Congo [13], [14]	1998–2000	Multiplelineages	154 (128)	Infections related to mining; multiple virus lineages; short transmission chains in families
Angola [15], [16]	2004/2005	Angola	252 (227)	Unknown origin; cases linked to Uige hospital and included high number of paediatric cases
Uganda [5]	2007	Multiple lineages	4 (1)	Infections related to visits of a mine (Kitaka Cave)
United States [17]	2008	n.d.	1 (0)	Unknown origin; infection related to visit of cave in western Uganda
The Netherlands [18]	2008	n.d.	1 (1)	Unknown origin; infection related to visit of cave in western Uganda

[7]–[18]  =  numbers in reference list; n.d.  =  not defined. To date, a total approximately 450 cases of MHF have been officially reported with case fatality rates in outbreaks ranging from ∼22–90%.

In March 2005, the National Microbiology Laboratory (NML) of the Public Health Agency of Canada (PHAC) offered assistance to the World Health Organization (WHO) as a partner of the ‘Global Outbreak Alert & Response Network’ (GOARN) (http://www.who.int/csr/outbreaknetwork/en/) for the MHF outbreak in Angola. Under GOARN, a Mobile Laboratory Unit (MLU) was deployed to Uige, the epicentre of the outbreak, to assist in clinical management and epidemiological surveillance with MARV-specific and limited differential diagnostic capacity. Here we discuss the usefulness of this latest response capacity for the management of viral hemorrhagic fever outbreaks.

## Methods

### Laboratory operation

**Figure 1:**
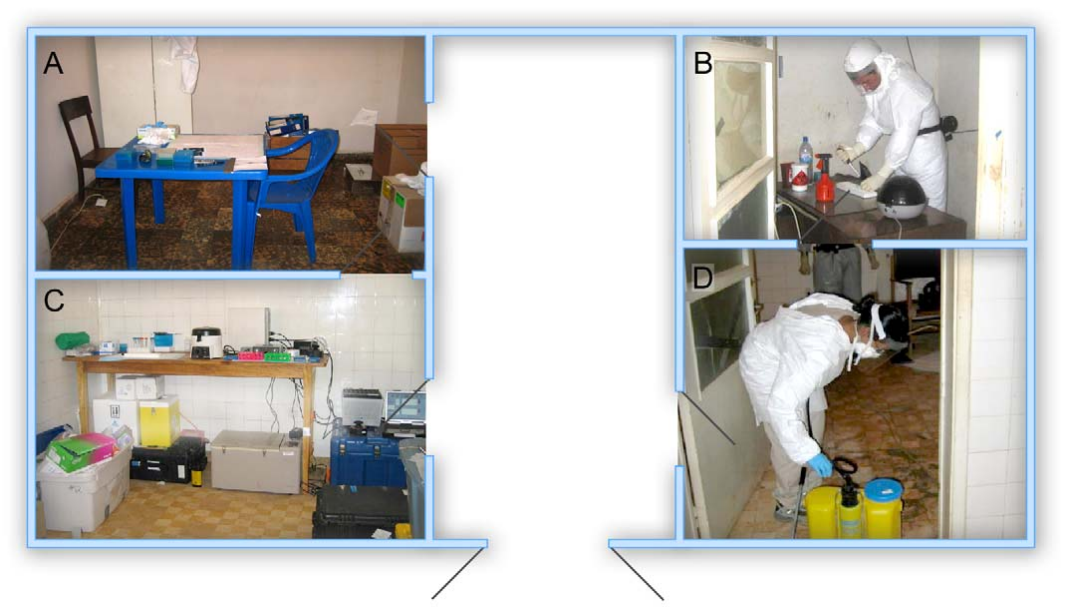
Laboratory set up and procedures. Laboratory space was made available to us in the Paediatric Ward of the Uige Provincial Hospital. Four rooms were used for the laboratory set up to ensure isolation of infectious work from other activities and to separate PCR assay steps to minimize contamination. (A) Room for RT-PCR master mix preparation; (B) room for sample inactivation; (C) room for RNA extraction and real-time RT-PCR; (D) room for PPE donning and disinfection.

Laboratory space was made available for the MLU in the Paediatric Ward of the Uige Provincial Hospital (Figure 1). Four rooms were used for the laboratory set up to ensure isolation of infectious work from other activities and to separate PCR assay steps to minimize contamination. Two rooms were located on one side of a central hallway; the smaller of the two rooms was accessible by a single door and had no windows or other opening and was utilized for infectious work (‘hot room’). The anteroom to this room was used for the preparation for entry to the infectious room and the subsequent disinfection of the worker following infectious work. Opposite these rooms were two additional rooms; one was used for RNA extraction and running the Q-RT-PCR and the other room was utilized as a ‘clean room’ for master mix preparation. Reagents and the laboratory team (2–3 members) were replaced every three weeks; in total NML deployed six teams to Angola to cover the period of April 1 to June 27, 2005.

### Sample collection

Clinical samples were collected by personnel wearing personal protective equipment (PPE) including a surgical mask, cap, shield or goggles, gown, apron, gloves (two pairs) and boots. Swab samples (nasal and oral) were collected using cotton tipped applicators (AMG Medical, VWR, Mississauga, ON, Canada). Applicator tips were stored in 700 µl of Dulbecco's modified essential medium (DMEM) or phosphate buffered saline (PBS) supplemented with 5% bovine serum albumin (Invitrogen, Burlington, ON, Canada). Whole blood and serum samples were collected using EDTA and serum vacutainer tubes, respectively. For transport, tubes were sealed in plastic bags, surface disinfected with a 1% hypochlorite solution, sealed into a second bag or container and again surface disinfected. Collection of human specimens occurred on an outbreak response protocol and was approved by the local Scientific and Technical Coordination Committee in Uige, Angola.

### Sample handling and RNA extraction

Infectious specimens were manipulated in the field laboratory by personnel wearing Tyvek suits and HEPA filter-equipped powered air purifying respirators, in a room isolated and dedicated for this work (Figure 1). An aliquot (140 µl) was removed from each sample and inactivated by adding 560 µl of the guanidine thiocyanate lysis buffer AVL. The sample tubes were submerged in 1% hypochlorite solution for 10 minutes and released from the infectious area. All further work was performed with PPE as outlined above. For RNA isolation we used the QIAamp Viral RNA mini kit (Qiagen, Mississauga, ON, Canada). All waste material was treated with 1% hypochlorite solution and incinerated on the same day. Two separate sample aliquots were prepared for transportation to the reference laboratory in Luanda operated by the Special Pathogens Branch of the US Centers for Disease Control and Prevention (US-CDC). Remaining samples were forwarded to the National Institute for Communicable Diseases (NCID), Sandringham, South Africa, and finally shipped to the US-CDC (Atlanta) or NML (Winnipeg) for further testing. Transportation was carried out in compliance with International Air Transport Association (IATA) regulations after prior approval by the appropriate national authorities of the sending or receiving countries.

### RT-PCR diagnostic assays

Initially, two quantitative real-time PCR (Q-RT-PCR) assays were used that targeted regions of the polymerase (L) [MARVLF-TTATTGCATCAGGCTTCTTGGCA, MARVLR–GGTATTAAAAAATGCATCCAA (AY358025; bp.13321–133517)] and the glycoprotein (GP) genes [MARVGPF–AAAGTTGCTGATTCCCCTTTGGA, MARVGPR–GCATGAGGGTTTTGACCTTGAAT (AY358025; bp.6131–6355)]. Later, an assay that targeted the nucleoprotein (NP) gene [MARVNPF–TGAATTTATCAGGGATTAAC, MARVNPR–GTTCATGTCGCCTTTGTAG (AY358025; bp.967–1146)] was used in place of the GP assay. The switch to an NP target was the result of testing that indicated this target was potentially more sensitive and provided a more distinct melting curve which simplified interpretation. MARV RNA was detected using the Lightcycler RNA Amplification SYBR Green I kit (Roche, Laval, PQ). Briefly, 5 µl of RNA was added to 20 µl of master mix containing 1X SYBR Green I mix, 5 mM MgCl_2_, 0.6 µM forward and reverse primers and 0.5 µl of the enzyme mix. Q-RT-PCR assays were run on Smartcycler thermocyclers (Cepheid, Sunnyvale, CA). A reverse transcriptase step at 50°C for 20 minutes and a 2 minute inactivation step at 94°C were followed by 40 cycles at 94°C for 15 seconds, 50°C for 30 seconds and 72°C for 30 seconds where a single acquisition point was taken. Melt curve analysis was performed to confirm the identity of amplification products. Samples were considered positive if they produced melting point confirmed amplification products in two assays. Amplification products were later confirmed by sequencing at NML (Winnipeg).

## Results

**Figure 2:**
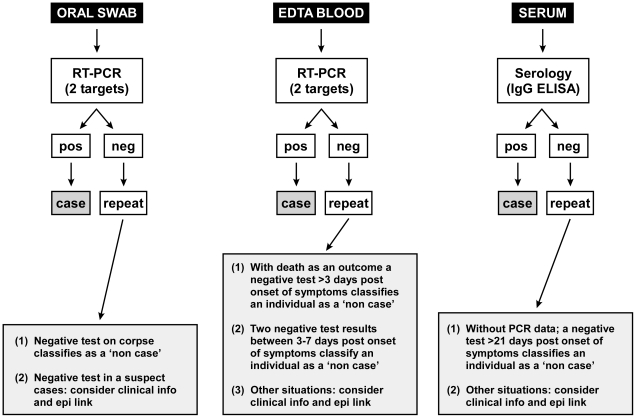
Diagnostic algorithm. Key: pos  =  positive; neg  =  negative.

The algorithm for the laboratory testing and the rational for positive/negative test results are presented in Figure 2. Overall, the MLU tested 620 clinical specimens from 388 patients/individuals over an operation period of 88 days. The clinical specimens included mainly oral swabs and EDTA blood/serum samples; the remainder consisted of nasal and conjunctival swabs and breast milk. The sample source and test results of individuals tested are presented in Table 2.

**Table 2:** MLU Sample source and test results.

Case sample source	Total persons tested	Persons positive/negative
Blood samples only	52	7/45
Blood and swab samples	116	28/88
Swab samples only	220	95/125
**Total**	**388**	**130 (258)**

Field lab processed 620 patient samples from 388 individuals during the 88 day operation of the lab. For the majority of cases only swab samples were available.

**Figure 3:**
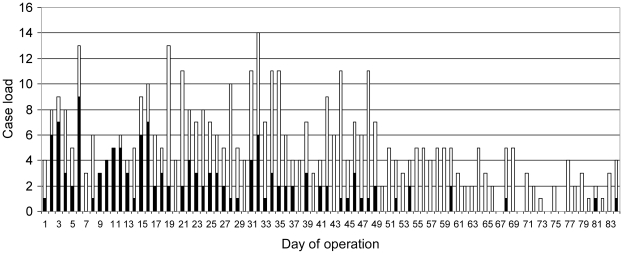
Field MARV diagnostic lab at Uige, Angola 2005; daily case load and positive sample detection. The height of each bar represents the total daily case load for the lab with the positive cases indicated by the solid portion.

**Figure 4:**
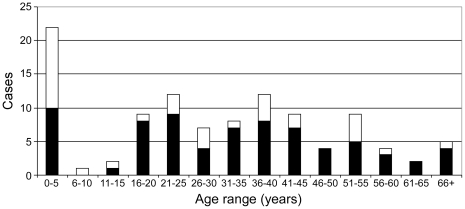
Age and gender distribution of positive cases. Positive cases identified during the operation of the field lab are shown separated by age and gender (female ▪ and male □). The distribution of positive cases demonstrates a higher proportion of females (68%) and children, 0 to 5 years, (21%) among the infected individuals.

The daily case load of the MLU fluctuated, with the number of individuals analyzed per day varying between 0 and 14 (Figure 3). This analysis often included multiple samples per individual on a single day and serial surveillance sampling of suspect and confirmed cases. The age and sex distribution of individuals tested were slightly shifted towards females (68%) and the younger age groups, in particular children under the age of 5 years (by far the largest single age group at 21%). The distribution of positive cases clearly demonstrated a larger proportion of females and children among the infected individuals (Figure 4).

**Figure 5:**
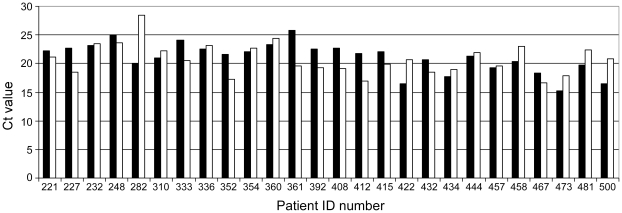
Cycle threshold values for paired blood and swab samples. Cases where whole blood (▪) and swab (□) samples were available for testing the same day are shown. Viral loads from both sample sources were comparable and do not consistently indicate one sample source as more suitable for viral load determination.

A comparison of detection of MARV from oral swabs and EDTA blood was performed on 63 individuals from whom both specimen types were available from the same day. Both samples sources yielded identical test results in 98.5% of the individuals with roughly 33% positive and 66% negative for MARV. Cycle threshold (Ct) values for most paired samples did not differ markedly indicating similar viral loads in both specimen sources (Figure 5). Testing on some patients did provide disparate results for blood and swab samples but test results were identical even in these instances. Similarly, for 12 individuals, both oral and nasal swabs specimens were collected which resulted in identical test results and no significant differences in Ct values for the positives. Additionally, 3 breast milk specimens from laboratory-confirmed female MHF cases were analyzed and shown to be positive for MARV (data not shown).

We did not experience any evidence for PCR contamination during the entire operation. All controls produced the expected positive and negative results. Nevertheless, all samples tested in Uige were subsequently shipped to Luanda for confirmation at a US-CDC established biosafety level 3 (BSL3) laboratory using a real-time PCR hybridization assay targeting the matrix protein (VP40) gene, an antigen capture enzyme-linked immunorsorbent (ELISA) assay and antibody (IgM and IgG) detection ELISAs [16]. Overall, the reference laboratory confirmed test results of the MLU in 97.5% of all specimens analyzed and in all but one case. The high concordance between field and reference laboratory results supported the on-site report of the MLU results to the ward and the surveillance teams, allowing a turn-around time of <4 hours from sample receiving to laboratory diagnosis. After closing the MLU, further clinical specimens were shipped to Winnipeg for diagnosis via Luanda (US-CDC) and Sandringham (NCID). Eventually, all specimens were shipped to the BSL4 laboratories in Atlanta and/or Winnipeg for additional analysis. Sequence analysis of all amplified products and of several virus isolates obtained at the US-CDC [16] and NML (authors, unpublished data) demonstrated a high degree of conservation indicating a single or very few introductions into the community, with subsequent human-to-human transmission.

Differential diagnostic testing was only performed for malaria (*Plasmodium spp*.) using a real time PCR assay targeting the ssuRNA gene [21]. Test results for 19 individuals demonstrated two groups of patients, mild or asymptomatic (Ct values >20) and symptomatic individuals (Ct values <20), based on parasitemia levels (data not shown). The value of this diagnostic tool needs to be further evaluated.

## Discussion

Under current filoviral hemorrhagic fever outbreak operation protocols several activities are undertaken where accurate and rapid diagnostic testing can have significant impact:

- Suspected patients are to be admitted to the isolation ward and managed by watching clinical progression and response; discharging those who responded quickly to empiric treatment, and following the rest until they recover or succumb to disease.
- Contact tracing of suspected cases requiring daily monitoring of family members and close contacts by field teams dedicated to this essential outbreak control function.
- Unexplained deaths are routinely treated as possible cases and bodies are buried safely to limit further spread but denying the family their customary burial practices.
- Those fortunate to recover can be shunned by their family, if any remain, or the community at large due to the fear they can act as a new source of infection.

To obtain diagnostic testing, specimens have normally been shipped to an international reference laboratory such as the Institut de recherche pour le développement (IRD), Franceville, Gabon; NCID in Sandringham, South Africa; or the US-CDC in Atlanta, United States resulting in a significant delay (days to weeks due to shipment issues) in laboratory diagnosis with limited or no benefit for acute case patient or outbreak management [22]–[24]. Therefore, such operation protocols require a fairly large infrastructure, longer hospitalization periods, and more staff and consequently increase resources and exposure risks. An MLU, providing testing results in a 4 hour turn around, can be an integral part of the outbreak response and simplify lessen many of the efforts needed to quickly contain and control the outbreak.

Laboratory testing of a symptomatic individuals during triage will allow the team to quickly assess if the person is a case or not. Confirmed cases can be appropriately isolated and supportive care initiated. Symptomatic individuals with negative test results can be maintained separate from confirmed cases either by releasing to another ward or kept in an observation ward for follow up testing or discharging. In Uige, and to a lesser extent also at previous outbreak locations, the isolation ward was largely unacceptable to the local population and significant resistance was present to have family members admitted [15], [25]. However a positive test result for MARV was normally sufficient to convince people of the necessity for admission to the ward. Isolating only those individuals who require it will reduce the infrastructure needed for isolation, minimize the hospitalization time for non-cases, reduce the number of staff and consequently reduce the risk of exposure for both staff and non-cases.

Cases that can be confirmed or excluded by laboratory testing can significantly contribute to one of the most important outbreak control measures, contact tracing. The current protocols call for the follow-up of contacts of suspected cases for 21 consecutive days. The presence of a field laboratory can help to arrive at a rapid confirmed final diagnosis for each suspected case, thereby decreasing the burden of field teams who may frequently be conducting contract tracing of cases with uncertain diagnosis.

Testing in this outbreak found that oral swabs from severely ill or deceased patients were a suitable sample for MARV testing by Q-RT-PCR. This allowed the MLU to safely test samples from corpses of unknown cause and when possible, to release MARV-negative bodies to the family members for traditional and religious burial procedures, a sensitive issue with almost all local communities in endemic areas. The value of swabs from corpses for diagnostic purposes needs to be further evaluated in future outbreaks and perhaps confirmed by other technologies such as immunohistochemistry [26]. Post mortem RNA degradation might render a test falsely negative even so infectious Ebola virus has been detected in blood samples more than a month after blood draw and storage at room temperature [27]. Any test results should take clinical presentation and epidemiology into account.

A growing concern is the return of negative and convalescent patients to the community, which may increase with the implementation of more advanced case patient care and the perspective of treatment options in the future [2], [24], [28]. These people are often shunned by their families and neighbours and a timely negative test result as provided through the MLU may aid in their re-acceptance and safe re-introduction into the community.

In Angola, field diagnostic support was used for the first time in response to a MHF outbreak. Also the first time, the combined operation of a field and reference laboratory allowed for a unique evaluation of field diagnostic capacity under difficult circumstances and proved it to be accurate, efficient and safe in operation. There have been previous attempts to provide field laboratory diagnostics for outbreaks of Ebola hemorrhagic fever. In 1976 during the *Zaire ebolavirus* outbreak an immunofluorescence assay was used for acute case identification but the results were considered poor [29]. In 2000 during the Ebola outbreak (*Sudan ebolavirus*) the US-CDC operated a laboratory within the Gulu district at St. Mary's Lacor Hospital, Uganda, and used antigen capture and reverse transcription nested PCR (RT-PCR) to successfully diagnose infection in suspected patients [30]. In 2003 during the Ebola outbreak (*Zaire ebolavirus*) in Mbomo, The Republic of the Congo, NML together with partners from the IRD, Franceville, Gabon, and the Bundeswehr Institute of Microbiology, Munich, Germany, operated a small field laboratory under the lead of WHO using antigen capture and Q-RT-PCR to diagnose acute cases [31], [32].

In general, the usefulness of on-site laboratory support during filovirus outbreaks is not really questioned [2], [24], and, in particular, the positive experience from this MHF outbreak demonstrate that rapid turn-around RT-PCR diagnostics can clearly aid in surveillance and case management [15], [25]. PCR-based techniques can be prone to contamination resulting in false positive results. Here we used a technique that did not require opening of tubes largely reducing the risk of contamination. Other concerns have been raised towards the reliability of RT-PCR assays during early disease stages and for survivors in the early convalescent stage, the consequences of false-positive and false-negative results of RT-PCR assays could be dire to outbreak management [30]. Indeed, PCR-based assays, like other diagnostic tests, have weaknesses and do not produce reliable results under all circumstances. Therefore, independent, methodologically different, confirmatory assay such as antigen capture to support RT-PCR should be mandatory. However, nowadays most laboratories depend on PCR detection as their first and most rapid diagnostic methods and there are good reasons to support that choice [33]. If a confirmatory assay is not available or unsuccessful, alternatives for RT-PCR confirmation include sample re-extraction, a second clinical specimen and/or an assay with independent targets (Figure 2). Nevertheless, any diagnostics should not replace general and common sense precautions in case patient management and on-site laboratory diagnostics should be in close proximity to the ward allowing for continuous interaction between physicians/nurses and laboratory personnel [15], [25]. Importantly, during this field laboratory deployment, Q-RT-PCR proved to be very sensitive and reliable even in this challenging environment. Patient samples were positive in our testing beginning on the day of onset of symptoms but we did see that detection in swab samples could be delayed by a few hours when compared to blood this early in the course of illness.

The collection of appropriate clinical specimens for diagnostic testing has become an increasing problem during filovirus outbreaks. The reasons for this can include the lack of properly trained personnel, fear of personnel to apply invasive procedures, cultural objections to bleeding and any other invasive pre- and post mortem sampling procedure, and insufficient infrastructure for sampling and transportation [22], [24], [34]. In that respect, the MHF outbreak in Angola was not different from previous outbreaks. In particular, resistance in the community to bleeding and post mortem invasive procedures, such as cardiac puncture or liver biopsy, and the increasing resistance of aid personnel to apply invasive procedures in the field (community) made oral swabs the predominant clinical specimen available for testing. As demonstrated here on paired blood/oral swab samples, in general there was no significant difference in viral load between oral swabs and EDTA blood taken at the same time (Figure 5). This supported oral swabs as an alternative diagnostic specimen to blood. The few incidences when oral swabs were less suitable than EDTA blood related to early disease stage and early convalescent stage samples. Lower viral loads in oral swabs compared to EDTA blood, at these stages, are likely to explain this discrepancy. Additionally, there are inherent sampling variables associated with oral swabs (the technique and efficiency of swabbing; moisture level of the oral cavity) that are not present in a blood draw, which may also have a role in these differences. However, despite the fact that oral swabs seemed to have been an appropriate specimen source for laboratory testing during this outbreak, and oral/nasal swabs are valuable alternatives in cases of resistance in the affected population to invasive procedures, EDTA blood should remain the priority choice for a clinical specimen due to the longer period of detectable viremia, the suitability to serological-based testing, and the value for monitoring potential point of care therapies in future.

While this study is not a detailed epidemiologic study, brief mention of some of the data is warranted as it has not been yet published elsewhere. This MHF outbreak was unique in regards to its location, case number and case fatalities, but also showed a large proportion of paediatric cases and cases among woman in the child bearing ages [2], [24]. Since MARV, as Ebola virus, are usually transmitted through close contact with blood, secretions or excretions from infected patients, family members and medical personnel caring for patients or preparing bodies for burials are considered high risk exposure groups [2], [34]. It has been proposed that because women provide the majority of in-home care that this was the reason for the preponderance of cases in women [35]. Certainly women provide the majority of care for the children and since, especially early in the outbreak, children less than 5 years of age represented the largest single age group affected may also be reflective of this fact. Furthermore, the detection of MARV in breast milk during this outbreak indicates that breastfeeding might have played a role in virus transmission. This is supported by epidemiological data indicating transmission from infected mothers to their nursing babies followed, after death of the mothers, by virus transmission from the infected babies to wet nurses who subsequently infected their own nursing child (authors, unpublished observation). Other factors may have come into play including the alleged lack of appropriate infection control within the paediatric ward prior to the identification of the outbreak [36]. It is very unlikely that the predilection of women and young children represents a biological predisposition, given that the demographics of the outbreak changed through the course of the outbreak (i.e. early in the outbreak a very high percentage were paediatric cases whereas later cases became more evenly distributed by age), and yet the virus changed very little [16]. Without more detailed epidemiologic data, it remains unclear which of these transmission routes constituted significant mechanisms for virus spread in the Uige outbreak.

Offering differential diagnosis significantly increases the value of on-site diagnostics. This is much harder to achieve in the field and requires variable clinical specimen (in particular blood or stool), more manpower and more extensive and continuous supplies. At a minimum, malaria diagnostics (e.g. commercially available rapid dipstick tests) and diagnosis for severe gastrointestinal infections should be available. Proper case patient management including intravenous fluid administration would also require blood chemistry and haematology analysis, another capacity that needs to be considered for expansion of a field laboratory response capacity.

Most of what constitutes the MLU can be sourced from equipment that most reference laboratories would have access to from their normal compliment of equipment and supplies, however a dedicated MLU would likely require the investment of approximately $100 000 and a weekly deployment cost of $2000 for reagents and supplies. Logistic needs and costs during a mission can be best managed through a close working relationship with other organizations including the WHO and Médecins Sans Frontières (MSF). The greatest challenge to the operation of the MLU was the lack of consistent electrical power and our reliance on portable generators. This necessitated the use of battery backup systems for thermocyclers and did not allow for storage of samples or reagents at freezing temperatures as freeze-thaw cycles could not be avoided. Fortunately, all reagents were relatively stable at 4°C over a three week rotation period before replacement teams replenished the reagents. We were able to efficiently operate the MLU using teams of two members as the workload and workflow rarely justified additional staff. We have since recommended that teams of three be deployed to allow for rest and health issues.

In conclusion, the combined operation of a field and reference laboratory in this outbreak allowed for a unique evaluation of field diagnostic capacity under difficult circumstances. Rapid MARV-specific Q-RT-PCR was useful for triage and assessing the need for isolation. The quick turn-around of laboratory diagnosis on the basis of Q-RT-PCR assays significantly improved outbreak response efforts. Therefore we propose: *“On-site laboratory diagnosis should become a routine part of any future filovirus outbreak response as it provides all responders with valuable information to help minimize the extent and durations of these events”*.
